# Autophagy-lysosome networks in oligodendrocytes: a dynamic framework for myelin integrity

**DOI:** 10.3389/fcell.2026.1818512

**Published:** 2026-04-21

**Authors:** Isabel Jiménez-Ridruejo, Ainhoa Plaza-Zabala

**Affiliations:** 1 Department of Pharmacology, University of the Basque Country (UPV/EHU), Leioa, Spain; 2 Achucarro Basque Center for Neuroscience, Leioa, Spain

**Keywords:** autophagy, lysosome, membrane trafficking, myelin, myelin integrity, oligodendrocytes, proteostasis

## Abstract

Oligodendrocytes generate and maintain myelin, a highly specialized membrane system essential for efficient signal propagation and long-term white matter integrity. The extreme biosynthetic demands and unique architecture of myelinating oligodendrocytes place exceptional pressure on intracellular quality control pathways. In this context, autophagy-lysosome networks have emerged as central regulators of oligodendrocyte biology, operating beyond bulk degradation to coordinate membrane remodeling, organelle homeostasis, and selective protein turnover. Here, we synthesize current evidence demonstrating that autophagy is dynamically regulated across the oligodendrocyte lineage and fulfills stage-specific roles, from precursor maintenance and differentiation-associated remodeling to long-term maintenance of compact myelin. We highlight advances revealing selective autophagic degradation of myelin proteins, the spatial distribution of autophagy within myelinating cells, and functional interactions between autophagy, endo-lysosomal trafficking, and myelin integrity. We also discuss emerging concepts of functional heterogeneity within autophagy-related compartments and context-dependent routing of myelin-associated cargo. Finally, we outline key open questions that define current gaps in our understanding of oligodendroglial autophagy. By framing autophagy as an integrated regulatory network rather than a single pathway, this review provides a conceptual foundation for understanding myelin biology under physiological conditions and establishes a basis for future studies on white matter vulnerability.

## Introduction

The vertebrate brain relies on myelinated axons to sustain fast and energetically efficient signal propagation across long distances (Simons and Nave, 2015). This specialization is produced by oligodendrocytes, highly specialized glial cells that generate extensive multilamellar membrane structures to ensheath axons and support saltatory conduction (Simons and Nave, 2015). Beyond their classical role as passive insulators, oligodendrocytes represent a unique cellular type characterized by extreme membrane expansion, long-term structural stability, and high metabolic demands (Simons and Lyons, 2013; Nave and Werner, 2014).

To fulfill these functions, oligodendrocytes must coordinate the synthesis, trafficking, and maintenance of vast amounts of lipid- and protein-rich membrane while preserving cellular integrity over long periods of time (Simons and Nave, 2015). This challenge is particularly pronounced during the transition from precursor to myelinating oligodendrocyte, when cells undergo a sharp increase in biosynthetic activity to initiate myelin assembly (Aggarwal et al., 2011). Myelin architecture itself further adds complexity, as compact membrane domains coexist with cytoplasm-containing channels that enable local membrane turnover and metabolic support within the sheath (Snaidero et al., 2014).

**FIGURE 1:**
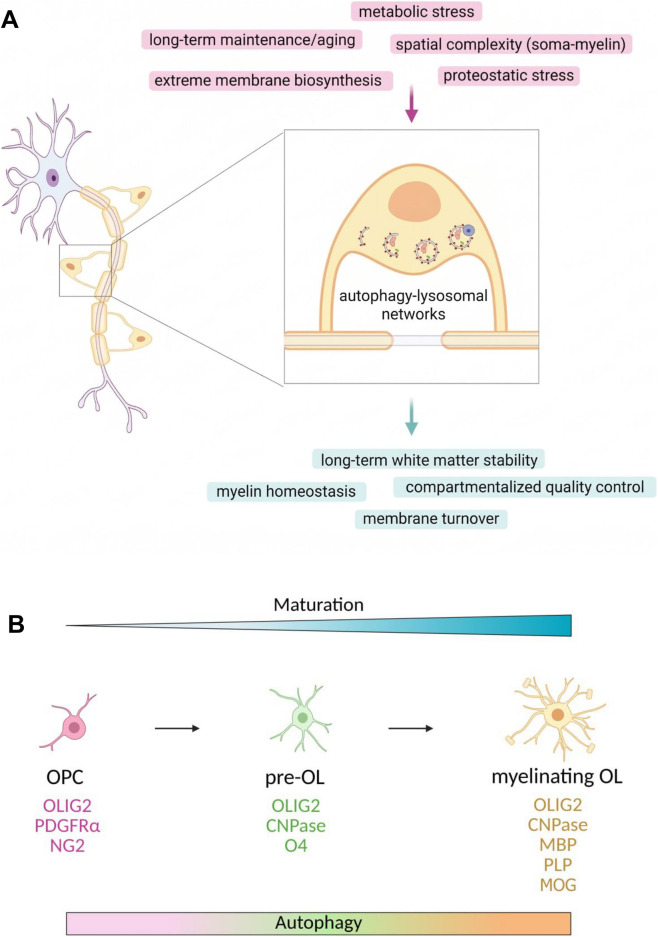
Autophagy-lysosome networks in oligodendrocyte biology. **(A)** Oligodendrocytes are highly specialized glial cells exposed to exceptional biological pressures, including extreme membrane biosynthesis, spatial complexity between soma and myelin sheath, proteostatic and metabolic stress, and the need for long-term maintenance across the lifespan. Autophagy-lysosome networks integrate these challenges by supporting compartmentalized quality control of proteins and organelles, membrane turnover, and myelin integrity, thereby contributing to long-term white matter stability. Instead of functioning solely as a degradative pathway, autophagy is depicted here as an adaptive regulatory network that sustains oligodendrocyte function. **(B)** Autophagy is presented as a dynamically regulated process across successive stages of oligodendrocyte lineage progression, schematically illustrated using representative markers (e.g. PDGFRalpha, O4, and MBP). Rather than operating uniformly across lineage states, autophagy adapts to the metabolic, biosynthetic, and structural demands associated with differentiation and myelin formation.

As a consequence, oligodendrocytes place an exceptional burden on intracellular organelles involved in energy production, protein and lipid biosynthesis, and membrane trafficking, including mitochondria, the endoplasmic reticulum, and lysosomes (Stadelmann et al., 2019). Both pre-myelinating and mature oligodendrocytes are therefore highly vulnerable to intracellular stress and perturbations in proteostasis and membrane homeostasis (Hughes and Stockton, 2021). These intrinsic vulnerabilities underscore the need for robust cellular quality control mechanisms and position autophagy-lysosome networks as central regulators of oligodendroglial function, survival, and long-term myelin integrity (Figure 1A).

## Autophagy-lysosome pathways: core principles

To sustain their extreme biosynthetic and structural demands, oligodendrocytes rely on fundamental intracellular quality control pathways, among which autophagy plays a central role. Autophagy comprises multiple lysosome-dependent pathways, including macroautophagy, chaperone-mediated autophagy (CMA), and microautophagy. CMA mediates the selective translocation of cytosolic proteins bearing KFERQ-like motifs directly across the lysosomal membrane via the receptor LAMP2A (Kaushik and Cuervo, 2018), whereas microautophagy involves the direct invagination of the endosomal or lysosomal membrane to engulf cytoplasmic material, including organelles and other cellular components (Wang et al., 2023; Sakai et al., 2026). In this review, we primarily refer to macroautophagy (hereafter autophagy), the best characterized autophagic pathway in oligodendrocytes, involving vesicular cargo sequestration and subsequent lysosomal degradation.

Autophagy is an evolutionarily conserved process through which cytoplasmic material is enclosed within double-membrane autophagosomes via the coordinated action of regulatory complexes, including the Unc-51-like kinase 1 (ULK1) complex, the class III phosphatidylinositol 3-kinase (PI3K) complex, and the autophagy-related (ATG) conjugation systems (Mizushima and Komatsu, 2011; Klionsky et al., 2021). These vesicles subsequently fuse with lysosomes, enabling cargo degradation and recycling.

Autophagosome maturation is tightly integrated with the broader endo-lysosomal system, reflecting the functional continuity between autophagic and endocytic trafficking pathways (Birgisdottir Å and Johansen, 2020). This integration is particularly relevant in brain cells, where compartmental organization, long-distance transport, and membrane dynamics impose strong constraints on lysosome-dependent processing mechanisms (Stavoe and Holzbaur, 2019).

Microtubule-associated protein 1 light chain 3 (LC3) and sequestosome-1 (SQSTM1/p62) are commonly used markers to monitor autophagy, reflecting autophagosome formation and cargo recruitment, respectively. However, their interpretation requires careful consideration, as static measurements do not necessarily capture autophagy flux (Klionsky et al., 2021; Plaza-Zabala and Sierra, 2024). Accordingly, LC3 is often evaluated alongside lysosomal markers such as lysosome-associated membrane protein-1 (LAMP1), lysosome-associated membrane protein-2 (LAMP2), or Cathepsin D, or in the presence of lysosomal inhibitors, to assess autophagic flux and cargo degradation (Mizushima and Komatsu, 2011; Plaza-Zabala et al., 2020; Plaza-Zabala and Sierra, 2024).

Importantly, autophagy-lysosome pathways do not operate solely as degradative mechanisms activated under stress, but function as constitutive regulatory systems that contribute to cellular organization, metabolic adaptation, and long-term cellular function.

## Autophagy-lysosome networks in neural cells: relevance for oligodendrocyte biology

Autophagy-lysosome pathways mediate intracellular quality control through the degradation of proteins, lipids and organelles, while also contributing to membrane turnover and recycling, metabolic adaptation, and large-scale cytoplasmic remodeling during cellular differentiation (Mizushima and Levine, 2010; Galluzzi et al., 2017). Such functions are particularly relevant in cells undergoing extensive morphological and functional reorganization and membrane expansion, including oligodendrocytes (Aggarwal et al., 2011; Snaidero et al., 2014).

In the Central Nervous System (CNS), autophagy-lysosome pathways operate under stringent spatial and temporal constraints imposed by long lifespan, structural complexity and the need for compartmentalized quality control mechanisms (Hara et al., 2006; Komatsu et al., 2006; Réu et al., 2017). Much of the current mechanistic understanding of these pathways in the brain derives from neuronal systems, where degradative processes are coordinated across highly polarized compartments through vesicular trafficking, cytoskeletal transport and local regulation at synapses (Nikoletopoulou et al., 2015; Stavoe and Holzbaur, 2019). These principles offer a useful conceptual basis for considering how autophagy may be organized in other neural cell types, including glia, despite their distinct structural and functional properties.

In oligodendrocytes, these constraints are further amplified by the need to support extensive membrane expansion and long-term myelin maintenance. Autophagic and lysosomal compartments are distributed not only within the soma but also along cytoplasmic channels embedded in the myelin sheath (Bankston et al., 2019; Aber et al., 2022), indicating that autophagy-lysosome pathways are spatially organized to support local functional demands. Within this context, autophagy appears closely integrated with the turnover of myelin-associated components and organelle quality control. Understanding how these networks operate across oligodendrocyte states may therefore help establish autophagy as a core component of oligodendroglial biology.

## Autophagy across the oligodendrocyte lineage: differentiation, survival and population dynamics

Autophagy is active throughout the oligodendrocyte lineage and is dynamically regulated as cells progress from proliferative oligodendrocyte precursor cells (OPCs) to pre-myelinating cells and, ultimately, mature myelinating oligodendrocytes (Figure 1B). Across this lineage, oligodendroglial cells undergo tightly coordinated transitions in proliferation, migration, cell-cycle exit and morphological differentiation (Bergles and Richardson, 2015), while later stages *in vivo* further involve axonal engagement, extensive membrane expansion and myelin compaction (Aggarwal et al., 2011; Snaidero et al., 2014). These differentiation states are commonly identified by the sequential expression of markers such as platelet-derived growth factor receptor α (PDGFRα) and neuron-glial antigen 2/chondroitin sulfate proteoglycan 4 (NG2/CSPG4) in OPCs, O4 and 2′-3′-cyclic nucleotide 3′-phosphodiesterase (CNP) in differentiating pre-oligodendrocytes, and myelin-associated proteins such as myelin basic protein (MBP), proteolipid protein (PLP) and myelin oligodendrocyte glycoprotein (MOG) in mature oligodendrocytes (Figure 1B). Much of our current understanding comes from *in vitro* differentiation models using primary OPCs, which have enabled the characterization of stage-specific changes in autophagic flux and lysosomal activity, while *in vivo* genetic studies have provided important complementary evidence supporting the role of these pathways in oligodendrocyte maturation and survival.

In proliferating OPCs, basal autophagic flux contributes to precursor maintenance and cell survival. Pharmacological inhibition of lysosomal acidification with bafilomycin A1 leads to LC3 puncta accumulation (Yazdankhah et al., 2021), consistent with ongoing autophagy under steady-state conditions. Likewise, molecular perturbation of core autophagy-related proteins such as ATG9 blocks autophagic flux and reduces both the proportion of proliferating OPCs and their viability (Yazdankhah et al., 2021), supporting a role for constitutive autophagy in precursor maintenance. Consistent with these observations, conditional genetic ablation of Atg5 in PDGFRα-expressing cells during the early postnatal period increased cell death within the PDGFRα positive population in the corpus callosum (Bankston et al., 2019), providing complementary *in vivo* evidence that autophagy supports OPC survival. Together, these findings support the idea that, at this stage, autophagy contributes to oligodendroglial precursor survival and maintenance by helping preserve intracellular quality control and metabolic balance in a rapidly dividing cell population.

As OPCs initiate differentiation, autophagy-lysosome networks become increasingly engaged. Multiple studies report a transient upregulation of autophagy flux during early differentiation (Bankston et al., 2019; Yazdankhah et al., 2021; Zhang et al., 2023), accompanied by increased vesicular trafficking (Bankston et al., 2019) and increased lysosomal content (Yazdankhah et al., 2021). These changes coincide with the onset of extensive biosynthetic programs required for morphological differentiation and membrane expansion. Imaging analyses further indicate that autophagic and lysosomal compartments increase during differentiation and, while remaining abundant in the soma, become progressively distributed along the increasingly elaborate oligodendroglial processes (Bankston et al., 2019; Aber et al., 2022). In neuron-oligodendrocyte co-cultures, autophagic vesicles were also observed in distal membrane domains associated with nascent myelin sheaths (Bankston et al., 2019), consistent with a spatial reorganization of autophagy during differentiation.

At later stages of oligodendrocyte differentiation, the contribution of autophagy appears to become more context-dependent. In culture models, autophagy interference can impair differentiation-associated outputs: dominant-negative inhibition of ATG5 reduced the proportion of O1-positive oligodendrocytes (Bankston et al., 2019), whereas ATG9 knockdown decreased MBP levels (Yazdankhah et al., 2021). Complementing these *in vitro* observations, inducible deletion of Atg5 in PDGFRα-expressing cells during the early postnatal period *in vivo*, reduced the proportion of CC1-positive mature oligodendrocytes in the corpus callosum at P9 (Bankston et al., 2019). However, other culture-based studies indicate that autophagy is not invariably required as a binary determinant of maturation, since conditional ablation of Atg5 in differentiating oligodendrocytes did not prevent the acquisition of mature morphology or the formation of myelin-like membrane sheets (Aber et al., 2022), highlighting that the impact of autophagy disruption depends on developmental context and experimental paradigm. Beyond differentiation-associated outputs, additional genetic evidence indicates that autophagy also shapes oligodendroglial survival and population dynamics during development.

Selective forms of autophagy also contribute to stage-specific remodeling during oligodendrocyte lineage progression. In particular, BCL2 interacting protein 3-like (BNIP3L/NIX)-dependent mitophagy has been described during oligodendrocyte differentiation, where it appears to support mitochondrial remodeling as cells transition toward a myelinating state (Yazdankhah et al., 2021). Notably, BNIP3L silencing did not affect OPC proliferation, but during differentiation it impaired mitochondrial clearance, altered mitochondrial and redox homeostasis, increased oligodendroglial cell death, and reduced MBP expression (Yazdankhah et al., 2021). In neuron-oligodendrocyte co-cultures, BNIP3L silencing also reduced the number of myelin-like segments formed along axons, further supporting a functional contribution of mitophagy to oligodendrocyte maturation and myelinogenic capacity (Yazdankhah et al., 2021). Consistent with this, *in vivo* Atg7 deficiency in NG2-positive progenitors has been associated with reduced oxidative phosphorylation in the corpus callosum (Chen et al., 2025), raising the possibility that impaired mitochondrial function may contribute to the long-term alterations in oligodendroglial populations observed after autophagy disruption.

At the population level, these changes translate into developmental outcomes that are highly sensitive to timing and lineage stage. Early postnatal deletion of Atg7 or Atg5 in PDGFRα-, OLIG2-, or CNP-expressing populations increases MBP-expressing oligodendrocyte number and ectopic myelination in the molecular layer of the cerebellum by modulating the Transcription Factor EB (TFEB)-p53 upregulated modulator of apoptosis (PUMA)-Bax/Bak apoptotic pathway in pre-myelinating oligodendrocytes (Sun et al., 2018; Zhang et al., 2023). By contrast, Atg5 deletion in PDGFRα-positive OPCs leads to defective myelination, oligodendrocyte loss, and early lethality (Bankston et al., 2019), highlighting that the outcome of autophagy disruption may vary according to developmental timing, the population targeted and the experimental setting. A similarly time-dependent pattern is observed after Atg7 deletion in NG2-positive progenitors: the density of PDGFRα-positive OPCs and CC1-positive mature oligodendrocytes is increased in the corpus callosum and hippocampus at 2 months of age, but reduced in both regions by 6 and 17 months (Chen et al., 2025). Together, these findings identify autophagy-lysosome networks as stage-dependent regulators of oligodendrocyte survival and population dynamics, with important implications for myelin development and long-term white matter stability.

## Functional coupling of autophagy-lysosome networks to myelin biogenesis, maintenance and white-matter function

In this section, we examine how autophagy-lysosomal networks contribute first to myelin biogenesis and membrane delivery, then to myelin protein quality control and turnover, and ultimately to white matter integrity and brain function.

### Lysosomal trafficking pathways in myelin biogenesis and oligodendrocyte membrane delivery

**FIGURE 2:**
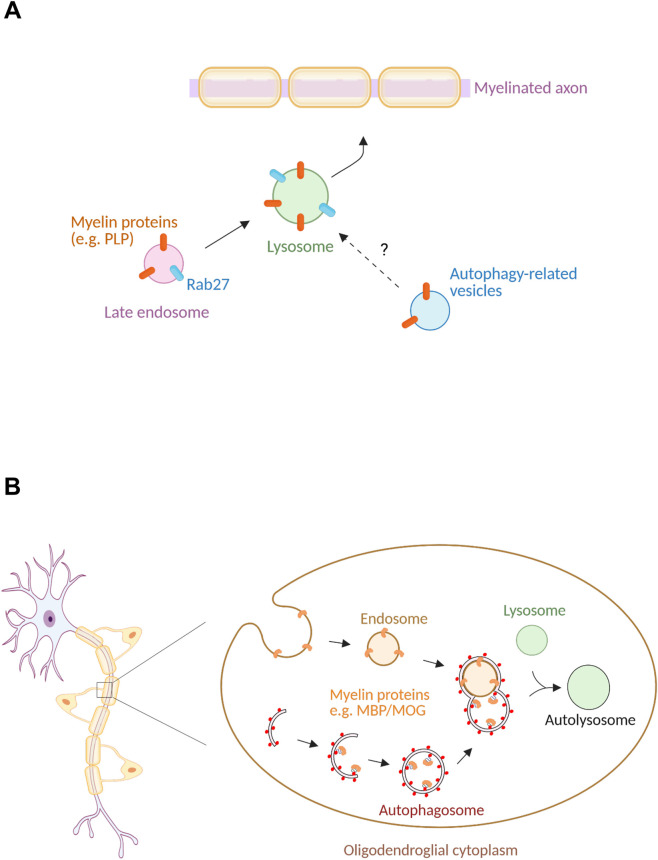
Autophagy-lysosome networks in myelin protein handling. **(A)** Conceptual model illustrating the role of late endosome-lysosome compartments as dynamic reservoirs for myelin-associated proteins within biosynthetic and trafficking pathways. Myelin proteins can accumulate within Rab27-positive late endosomal/lysosomal compartments, from which they may be mobilized through regulated trafficking pathways toward membrane delivery. The potential interaction between autophagy-related vesicles and lysosomal compartments is depicted to highlight emerging questions regarding cargo routing and membrane dynamics in oligodendrocytes. **(B)** Schematic representation of the functional coupling between endocytic trafficking, autophagosome formation, and lysosomal degradation in oligodendroglial cytoplasm. Myelin-associated proteins can be internalized through endosomal pathways or incorporated into autophagic vesicles, which subsequently fuse with lysosomes to form autolysosomes. This integrated trafficking network supports selective protein turnover and contributes to myelin integrity in mature oligodendrocytes.

Lysosomal pathways contribute to myelin biogenesis by regulating the trafficking, storage and surface delivery of myelin components (Figure 2A). Lysosomes comprise both degradative and secretory subpopulations, enabling them to support intracellular catabolism as well as regulated exocytosis (Andrews, 2000; Luzio et al., 2014). In oligodendrocytes, PLP localizes to late endosome/lysosome related compartments and can be mobilized from these reservoirs to the plasma membrane during myelin membrane formation, supporting a role for lysosome-related trafficking in membrane delivery (Feldmann et al., 2011; Shen et al., 2016).

Consistent with this model, Rab GTPases involved in late endosomal and lysosomal dynamics also contribute to myelin component trafficking and membrane delivery (Margiotta, 2023). In particular, Rab27b colocalizes with PLP and the lysosomal marker cathepsin D, and stimulation of calcium-dependent lysosomal exocytosis with ionomycin increases PLP surface levels in cultured oligodendrocytes, whereas Rab27b silencing reduces surface PLP and impairs myelin-like membrane sheet formation in neuron-oligodendrocyte co-cultures (Shen et al., 2016). Other trafficking regulators, such as Rab3a, have likewise been implicated in the membrane trafficking events that support oligodendrocyte morphological differentiation (Anitei et al., 2009) and regulate surface delivery of myelin proteins such as PLP (Schardt et al., 2009).

The importance of lysosomal function for myelin biology is further underscored by lysosomal storage disorders (LSDs) and related leukodystrophies, in which mutations affecting lysosomal enzymes or associated membrane proteins lead to substrate accumulation, altered lysosomal function and white matter abnormalities (Platt et al., 2018; Maegawa, 2019). For example, Krabbe disease, caused by deficiency of galactocerebrosidase (GALC), results in psychosine accumulation and severe dysmyelination, and has also been associated with autophagy defects (Del Grosso et al., 2019; Maegawa, 2019; Papini et al., 2023; Saldivia et al., 2024), whereas metachromatic leukodystrophy, caused by arylsulfatase A (ARSA) deficiency, leads to sulfatide storage, proteostasis defects, and progressive myelin degeneration (Maegawa, 2019; Lin and Ho, 2024). Although these disorders are mechanistically heterogeneous and their pathology is not necessarily restricted to oligodendrocytes, they collectively illustrate how disruption of lysosomal function can compromise myelin formation and stability. However, how lysosomal exocytosis and related trafficking pathways are coordinated with myelin component trafficking, storage and delivery, and how these pathways may interact with autophagy, remain to be defined.

### Autophagy in myelin protein quality control and turnover

Myelin must be maintained throughout life through continuous membrane remodeling and selective turnover of its components. Consistent with these demands, autophagy remains active in mature oligodendrocytes and operates within the highly specialized architecture of the myelin sheath (Figure 2B). Live-imaging studies in cultured oligodendrocytes demonstrate that autophagosomes can form and undergo acidification not only in the perikaryon but also within cytoplasm-containing myelin-like membrane regions (Bankston et al., 2019; Aber et al., 2022). Although autophagic vesicles can display bidirectional motility, most form and mature locally, consistent with a spatially distributed autophagy-lysosome system adapted to the geometry of differentiated oligodendrocytes (Bankston et al., 2019; Aber et al., 2022).

Biochemical and genetic evidence indicates that autophagy contributes to the selective turnover of myelin proteins. Autophagic vesicles isolated from the adult mouse brain contain multiple myelin associated proteins, including MBP, PLP, MOG, and MAG, in addition to their presence in myelin-enriched membrane fractions (Aber et al., 2022). Inhibition of autophagic flux with leupeptin in oligodendrocytes leads to the accumulation of these proteins and their association with autophagic and lysosomal markers such as LC3 and LAMP2 (Aber et al., 2022), supporting an active role for autophagy in myelin protein clearance.

Even for myelin proteins exposed at the extracellular surface, turnover requires regulated internalization and delivery to intracellular degradative compartments. In this context, proteins such as MOG are internalized through specialized endocytic pathways and diverted away from recycling routes toward autophagosome-lysosome degradation via amphisome formation (Aber et al., 2022). These findings highlight a tight coupling between endocytosis, autophagy, and lysosomal degradation in sustaining myelin integrity.

*In vivo* studies have established MBP as a prominent substrate for selective autophagy. Co-immunoprecipitation analyses reveal interactions between MBP and selective autophagy machinery, including SQSTM1/p62, autophagy-linked FYVE protein (ALFY), and LC3 (Aber et al., 2022). Consistent with this, conditional deletion of Atg5 or Atg7 in NG2-positive oligodendrocyte precursors and CNP-expressing (pre)-myelinating oligodendrocytes results in the accumulation of detergent-insoluble MBP aggregates enriched in the autophagy adaptor SQSTM1/p62 (Aber et al., 2022; Chen et al., 2025), a finding that aligns with the known aggregation propensity of this intrinsically disordered protein. Importantly, MBP aggregation becomes more pronounced with age (Chen et al., 2025) and is further exacerbated by autophagy impairment across distinct stages of the oligodendrocyte lineage (Aber et al., 2022; Chen et al., 2025), reinforcing the concept that selective autophagy constitutes a central mechanism for long-term protein quality control within myelin membranes.

### Impact of oligodendroglial autophagy on white matter integrity and brain function

Consistent with these roles in myelin protein turnover and quality control, genetic disruption of autophagy at distinct stages of the oligodendrocyte lineage leads to structural abnormalities of the myelin sheath. Whereas Atg7 deletion in NG2-expressing progenitors causes myelin defects detectable by 6 months of age (Chen et al., 2025), constitutive Atg7 deletion in CNP-expressing (pre)-myelinating oligodendrocytes and inducible Atg5 deletion in PLP-expressing oligodendrocytes in adulthood result in myelin abnormalities that become evident in aged mice (Aber et al., 2022; Ktena et al., 2025). Together, these findings indicate that autophagy is required throughout the oligodendrocyte lineage for the lifelong maintenance of myelin integrity.

Disruption of oligodendroglial autophagy can also have broader consequences for white matter integrity and brain function. In aged mice, autophagy deficiency in (pre)-myelinating oligodendrocytes not only causes myelin abnormalities but is also associated with increased microglial density and reactivity (Ktena et al., 2025), motor impairment (Aber et al., 2022), and neurodegeneration (Aber et al., 2022; Ktena et al., 2025). These outcomes appear progressively with age when autophagy is disrupted in CNP-expressing (pre)-myelinating (Aber et al., 2022) or PLP-expressing mature oligodendrocytes (Ktena et al., 2025). These data support the view that oligodendroglial autophagy is required to preserve white matter integrity and restrain inflammatory and neurodegenerative deterioration during aging.

Conversely, enhancement of autophagy can exert protective effects on oligodendrocyte viability and myelin formation. In the Long-Evans Shaker (LES) rat, a dysmyelinating model caused by MBP deficiency, systemic activation of autophagy through intermittent fasting enhances myelin membrane extension and improves axonal ensheathment (Smith et al., 2013), suggesting that boosting autophagy-lysosome function may partially compensate for intrinsic defects in myelin biogenesis.

## Open questions and emerging directions

Despite significant progress in delineating the roles of autophagy-lysosome networks in oligodendrocyte biology, several fundamental questions remain unresolved. Addressing these gaps will be essential to fully understand how these pathways support myelin formation, maintenance, and long-term white matter integrity.

### How is autophagy selectively regulated across oligodendrocyte states?

Although autophagy is active throughout the oligodendrocyte lineage, the mechanisms that tune autophagic flux, cargo selectivity, and spatial organization at distinct developmental stages remain poorly defined. It is unclear how transcriptional, post-translational, and metabolic signals are integrated to adapt autophagy-lysosome networks to the rapidly changing demands of precursor cells, differentiating oligodendrocytes, and mature myelinating cells. Dissecting these regulatory layers will be critical to understand how autophagy supports both lineage progression and long-term cellular resilience.

### What determines cargo selectivity in myelin-associated autophagy?

Emerging evidence indicates that myelin proteins can be subject to selective autophagic degradation. For example, MBP undergoes adaptor-mediated selective autophagy involving SQSTM1/p62, ALFY and LC3 (Aber et al., 2022). However, the molecular determinants that target other myelin components to autophagosomes for degradation remain largely unknown. Whether cargo recognition relies on intrinsic protein features, post-translational modifications, adaptor proteins, or signals linked to neuronal activity along myelinated axons is an open question. Understanding how such selectivity is achieved may be critical to explain how oligodendrocytes maintain myelin proteostasis over time.

### Are autophagosomes functionally specialized in oligodendrocytes?

A key unresolved issue is whether autophagosomes involved in myelin protein handling are uniformly degradative or whether functionally distinct autophagic compartments exist in oligodendrocytes. It remains unknown whether molecular cues determine if myelin-associated cargo is routed toward lysosomal degradation or, under specific conditions, contributes to trafficking and delivery of proteins and lipids to the growing myelin membrane. Elucidating whether such context-dependent routing exists would have important implications for understanding how oligodendrocytes coordinate myelin growth with quality control.

### How are autophagy-lysosome networks spatially organized within the myelin sheath?

The presence of autophagic and lysosomal compartments within cytoplasmic channels of the myelin sheath raises important questions regarding local versus centralized degradation. How autophagosomes form, mature, and fuse with lysosomes across distant subcellular domains, and how these processes are coordinated with vesicular trafficking and cytoskeletal transport, remain largely unexplored. Elucidating the spatial logic of autophagy in myelinating oligodendrocytes will be key to understanding how quality control is maintained within an extreme cellular architecture.

### How does autophagy interface with lysosomal exocytosis and membrane trafficking during myelin biogenesis?

While studies in cultured oligodendrocytes have implicated lysosomal pathways in the regulated trafficking and delivery of myelin proteins such as PLP from endo-lysosomal compartments to the plasma membrane (Anitei et al., 2009; Schardt et al., 2009; Shen et al., 2016), the extent to which autophagy intersects with secretory lysosome pools during myelin biogenesis remains unclear. Whether distinct autophagy-related compartments contribute directly to the supply of membrane components for myelin growth, or instead influence myelin biogenesis indirectly by regulating autophagic flux and the availability and composition of endo-lysosomal trafficking intermediates, represents an important area for future investigation.

### What is the relationship between autophagy, cell survival, and oligodendrocyte population dynamics?

Genetic studies reveal that perturbing autophagy at different developmental stages can lead to divergent outcomes, including increased oligodendrocyte numbers and ectopic myelination following early postnatal autophagy disruption (Zhang et al., 2023), or impaired myelination, oligodendrocyte loss, and early lethality when autophagy is perturbed at slightly later stages (Bankston et al., 2019). These findings build on the concept that oligodendrocyte number and spatial distribution are actively regulated during development (Marques et al., 2016), and highlight a context-dependent role for autophagy in balancing survival, apoptosis, and population size. Defining how autophagy interfaces with developmental cell death pathways, including apoptosis, and how this balance is modulated temporally and regionally remains an open challenge (Sun et al., 2018; Zhang et al., 2023).

### Can autophagy-lysosome pathways be leveraged to enhance myelin resilience?

Finally, the observation that stimulating autophagy can ameliorate myelin defects in specific dysmyelinating contexts raises the possibility that pharmacological modulation of autophagy-lysosome networks could be leveraged to enhance oligodendrocyte resilience and myelin stability (Smith et al., 2013). However, given the stage-dependent and multifunctional roles of autophagy, a deeper mechanistic understanding will be required to identify when and how these pathways can be targeted therapeutically without disrupting essential homeostatic functions.

Together, these open questions emphasize that autophagy-lysosome networks are not ancillary pathways in oligodendrocytes, but central regulators of myelin dynamics and white matter integrity. Future work integrating genetic, imaging, and systems-level approaches, together with targeted perturbation strategies, will be crucial to resolve how these networks operate across space, time, and cellular state, and to determine how they can be rationally targeted to enhance myelin resilience.

## Conclusion

Autophagy-lysosome networks constitute a central regulatory system in oligodendrocytes, dynamically tuned to support myelin formation, maintenance, and long-term white matter integrity. Framing autophagy as an integrated network rather than a single pathway provides a conceptual lens to understand how oligodendrocytes sustain extreme membrane architectures across the lifespan, and highlights opportunities to interrogate and modulate these networks to enhance myelin resilience.

## References

[B1] Aber E. R. Griffey C. J. Davies T. Li A. M. Yang Y. J. Croce K. R. (2022). Oligodendroglial macroautophagy is essential for myelin sheath turnover to prevent neurodegeneration and death. Cell Rep. 41, 111480. 10.1016/j.celrep.2022.111480 36261002 PMC9639605

[B2] Aggarwal S. Yurlova L. Simons M. (2011). Central nervous system myelin: structure, synthesis and assembly. Trends Cell Biol. 21, 585–593. 10.1016/j.tcb.2011.06.004 21763137

[B3] Andrews N. W. (2000). Regulated secretion of conventional lysosomes. Trends Cell Biol. 10, 316–321. 10.1016/s0962-8924(00)01794-3 10884683

[B4] Anitei M. Cowan A. E. Pfeiffer S. E. Bansal R. (2009). Role for Rab3a in oligodendrocyte morphological differentiation. J. Neurosci. Res. 87, 342–352. 10.1002/jnr.21870 18798275

[B5] Bankston A. N. Forston M. D. Howard R. M. Andres K. R. Smith A. E. Ohri S. S. (2019). Autophagy is essential for oligodendrocyte differentiation, survival, and proper myelination. Glia 67, 1745–1759. 10.1002/glia.23646 31162728

[B6] Bergles D. E. Richardson W. D. (2015). Oligodendrocyte development and plasticity. Cold Spring Harb. Perspect. Biol. 8, a020453. 10.1101/cshperspect.a020453 26492571 PMC4743079

[B7] Birgisdottir Å B. Johansen T. (2020). Autophagy and endocytosis - interconnections and interdependencies. J. Cell Sci. 133, jcs228114. 10.1242/jcs.228114 32501285

[B8] Chen H. Yang G. Xu D. E. Du Y. T. Zhu C. Hu H. (2025). Autophagy in oligodendrocyte lineage cells controls oligodendrocyte numbers and Myelin integrity in an age-dependent manner. Neurosci. Bull. 41, 374–390. 10.1007/s12264-024-01292-1 39283565 PMC11876512

[B9] Del Grosso A. Angella L. Tonazzini I. Moscardini A. Giordano N. Caleo M. (2019). Dysregulated autophagy as a new aspect of the molecular pathogenesis of Krabbe disease. Neurobiol. Dis. 129, 195–207. 10.1016/j.nbd.2019.05.011 31108173

[B10] Feldmann A. Amphornrat J. Schönherr M. Winterstein C. Möbius W. Ruhwedel T. (2011). Transport of the major myelin proteolipid protein is directed by VAMP3 and VAMP7. J. Neurosci. 31, 5659–5672. 10.1523/JNEUROSCI.6638-10.2011 21490207 PMC6622839

[B11] Galluzzi L. Baehrecke E. H. Ballabio A. Boya P. Bravo-San Pedro J. M. Cecconi F. (2017). Molecular definitions of autophagy and related processes. EMBO J. 36, 1811–1836. 10.15252/embj.201796697 28596378 PMC5494474

[B12] Hara T. Nakamura K. Matsui M. Yamamoto A. Nakahara Y. Suzuki-Migishima R. (2006). Suppression of basal autophagy in neural cells causes neurodegenerative disease in mice. Nature 441, 885–889. 10.1038/nature04724 16625204

[B13] Hughes E. G. Stockton M. E. (2021). Premyelinating oligodendrocytes: mechanisms underlying cell survival and integration. Front. Cell Dev. Biol. 9, 714169. 10.3389/fcell.2021.714169 34368163 PMC8335399

[B14] Kaushik S. Cuervo A. M. (2018). The coming of age of chaperone-mediated autophagy. Nat. Rev. Mol. Cell Biol. 19, 365–381. 10.1038/s41580-018-0001-6 29626215 PMC6399518

[B15] Klionsky D. J. Abdel-Aziz A. K. Abdelfatah S. Abdellatif M. Abdoli A. Abel S. (2021). Guidelines for the use and interpretation of assays for monitoring autophagy (4th edition)(1). Autophagy 17, 1–382. 10.1080/15548627.2020.1797280 33634751 PMC7996087

[B16] Komatsu M. Waguri S. Chiba T. Murata S. Iwata J. Tanida I. (2006). Loss of autophagy in the central nervous system causes neurodegeneration in mice. Nature 441, 880–884. 10.1038/nature04723 16625205

[B17] Ktena N. Spyridakos D. Georgilis A. Kalafatakis I. Thomoglou E. Kolaxi A. (2025). Disruption of oligodendroglial autophagy leads to myelin morphological deficits, neuronal apoptosis, and cognitive decline in aged mice. Glia 73, 1383–1397. 10.1002/glia.70012 40105013 PMC12121467

[B18] Lin D. S. Ho C. S. (2024). Emerging role of ubiquitin proteasome system and autophagy in pediatric demyelinating leukodystrophies and therapeutic opportunity. Cells 13, 1873. 10.3390/cells13221873 39594621 PMC11593168

[B19] Luzio J. P. Hackmann Y. Dieckmann N. M. Griffiths G. M. (2014). The biogenesis of lysosomes and lysosome-related organelles. Cold Spring Harb. Perspect. Biol. 6, a016840. 10.1101/cshperspect.a016840 25183830 PMC4142962

[B20] Maegawa G. H. B. (2019). Lysosomal leukodystrophies lysosomal storage diseases associated with white matter abnormalities. J. Child. Neurol. 34, 339–358. 10.1177/0883073819828587 30757954 PMC6459700

[B21] Margiotta A. (2023). Role of SNAREs and rabs in Myelin regulation. Int. J. Mol. Sci. 24, 9772. 10.3390/ijms24119772 37298723 PMC10254019

[B22] Marques S. Zeisel A. Codeluppi S. Van Bruggen D. Mendanha Falcão A. Xiao L. (2016). Oligodendrocyte heterogeneity in the mouse juvenile and adult central nervous system. Science 352, 1326–1329. 10.1126/science.aaf6463 27284195 PMC5221728

[B23] Mizushima N. Komatsu M. (2011). Autophagy: renovation of cells and tissues. Cell 147, 728–741. 10.1016/j.cell.2011.10.026 22078875

[B24] Mizushima N. Levine B. (2010). Autophagy in mammalian development and differentiation. Nat. Cell Biol. 12, 823–830. 10.1038/ncb0910-823 20811354 PMC3127249

[B25] Nave K. A. Werner H. B. (2014). Myelination of the nervous system: mechanisms and functions. Annu. Rev. Cell Dev. Biol. 30, 503–533. 10.1146/annurev-cellbio-100913-013101 25288117

[B26] Nikoletopoulou V. Papandreou M. E. Tavernarakis N. (2015). Autophagy in the physiology and pathology of the central nervous system. Cell Death Differ. 22, 398–407. 10.1038/cdd.2014.204 25526091 PMC4326580

[B27] Papini N. Todisco R. Giussani P. Dei Cas M. Paroni R. Giallanza C. (2023). Impaired autophagy in Krabbe disease: the role of BCL2 and Beclin-1 phosphorylation. Int. J. Mol. Sci. 24, 5984. 10.3390/ijms24065984 36983059 PMC10051825

[B28] Platt F. M. D'azzo A. Davidson B. L. Neufeld E. F. Tifft C. J. (2018). Lysosomal storage diseases. Nat. Rev. Dis. Prim. 4, 27. 10.1038/s41572-018-0025-4 30275469

[B29] Plaza-Zabala A. Sierra A. (2024). Studying autophagy in microglia: overcoming the obstacles. Methods Mol. Biol. 2713, 45–70. 10.1007/978-1-0716-3437-0_3 37639114

[B30] Plaza-Zabala A. Sierra-Torre V. Sierra A. (2020). Assessing autophagy in microglia: a two-step model to determine autophagosome formation, degradation, and net turnover. Front. Immunol. 11, 620602. 10.3389/fimmu.2020.620602 33584716 PMC7878397

[B31] Réu P. Khosravi A. Bernard S. Mold J. E. Salehpour M. Alkass K. (2017). The lifespan and turnover of microglia in the human brain. Cell Rep. 20, 779–784. 10.1016/j.celrep.2017.07.004 28746864 PMC5540680

[B32] Sakai Y. Behrends C. Debnath J. Izumi M. Jenny A. Molinari M. (2026). Microautophagy: current understanding of its molecular mechanisms and functions. Autophagy Rep. 5, 2626661. 10.1080/27694127.2026.2626661 41756808 PMC12934144

[B33] Saldivia N. Heller G. Zelada D. Whitehair J. Venkat N. Konjeti A. (2024). Deficiency of galactosyl-ceramidase in adult oligodendrocytes worsens disease severity during chronic experimental allergic encephalomyelitis. Mol. Ther. 32, 3163–3176. 10.1016/j.ymthe.2024.06.035 38937968 PMC11403238

[B34] Schardt A. Brinkmann B. G. Mitkovski M. Sereda M. W. Werner H. B. Nave K. A. (2009). The SNARE protein SNAP-29 interacts with the GTPase Rab3A: implications for membrane trafficking in myelinating glia. J. Neurosci. Res. 87, 3465–3479. 10.1002/jnr.22005 19170188

[B35] Shen Y. T. Gu Y. Su W. F. Zhong J. F. Jin Z. H. Gu X. S. (2016). Rab27b is involved in lysosomal exocytosis and proteolipid protein trafficking in oligodendrocytes. Neurosci. Bull. 32, 331–340. 10.1007/s12264-016-0045-6 27325508 PMC5563785

[B36] Simons M. Lyons D. A. (2013). Axonal selection and myelin sheath generation in the central nervous system. Curr. Opin. Cell Biol. 25, 512–519. 10.1016/j.ceb.2013.04.007 23707197

[B37] Simons M. Nave K. A. (2015). Oligodendrocytes: myelination and axonal support. Cold Spring Harb. Perspect. Biol. 8, a020479. 10.1101/cshperspect.a020479 26101081 PMC4691794

[B38] Smith C. M. Mayer J. A. Duncan I. D. (2013). Autophagy promotes oligodendrocyte survival and function following dysmyelination in a long-lived myelin mutant. J. Neurosci. 33, 8088–8100. 10.1523/JNEUROSCI.0233-13.2013 23637198 PMC4128639

[B39] Snaidero N. Möbius W. Czopka T. Hekking L. H. Mathisen C. Verkleij D. (2014). Myelin membrane wrapping of CNS axons by PI(3,4,5)P3-dependent polarized growth at the inner tongue. Cell 156, 277–290. 10.1016/j.cell.2013.11.044 24439382 PMC4862569

[B40] Stadelmann C. Timmler S. Barrantes-Freer A. Simons M. (2019). Myelin in the central nervous system: structure, function, and pathology. Physiol. Rev. 99, 1381–1431. 10.1152/physrev.00031.2018 31066630

[B41] Stavoe A. K. H. Holzbaur E. L. F. (2019). Autophagy in neurons. Annu. Rev. Cell Dev. Biol. 35, 477–500. 10.1146/annurev-cellbio-100818-125242 31340124 PMC6996145

[B42] Sun L. O. Mulinyawe S. B. Collins H. Y. Ibrahim A. Li Q. Simon D. J. (2018). Spatiotemporal control of CNS myelination by oligodendrocyte programmed cell death through the TFEB-PUMA axis. Cell 175, 1811–1826 e1821. 10.1016/j.cell.2018.10.044 30503207 PMC6295215

[B43] Wang L. Klionsky D. J. Shen H. M. (2023). The emerging mechanisms and functions of microautophagy. Nat. Rev. Mol. Cell Biol. 24, 186–203. 10.1038/s41580-022-00529-z 36097284

[B44] Yazdankhah M. Ghosh S. Shang P. Stepicheva N. Hose S. Liu H. (2021). BNIP3L-mediated mitophagy is required for mitochondrial remodeling during the differentiation of optic nerve oligodendrocytes. Autophagy 17, 3140–3159. 10.1080/15548627.2020.1871204 33404293 PMC8526037

[B45] Zhang T. Bhambri A. Zhang Y. Barbosa D. Bae H. G. Xue J. (2023). Autophagy collaborates with apoptosis pathways to control oligodendrocyte number. Cell Rep. 42, 112943. 10.1016/j.celrep.2023.112943 37543947 PMC10529879

